# Changing Dynamics in Liver Allocation: Donor and Recipient Traits Pre- and Post Acuity Circle Implementation

**DOI:** 10.7759/cureus.94206

**Published:** 2025-10-09

**Authors:** Yichen Fang, Jahnavi Nadella, Madison Yeager, Daniel Sculley, David Bagley, Noor Alyasiry, Raymond I Okeke, Justin Rehder, Mustafa Nazzal

**Affiliations:** 1 Department of Surgery, Division of General Surgery, SSM Health Saint Louis University Hospital, Saint Louis University School of Medicine, Saint Louis, USA; 2 Department of Surgery, Division of Abdominal Transplant, SSM Health Saint Louis University Hospital, Saint Louis University School of Medicine, Saint Louis, USA

**Keywords:** health disparities, liver, liver transplant, meld score, organ allocation

## Abstract

Background: The Acuity Circle (AC) allocation model for liver transplantation was implemented in 2020 to address geographic disparities in organ access. The policy improves equity and waitlist outcomes by prioritizing disease severity and reducing regional boundaries. This study reviews transplant metrics and donor and recipient characteristics in the pre- and post-AC implementation era.

Methods: Data from the National Standard Transplant Analysis and Research (STAR) file were analyzed for liver transplants performed between February 4, 2017, and February 3, 2023. Donor and recipient variables were assessed using chi-squared and t-tests. Geographic trends and changes in transplant volumes were analyzed.

Results: Following the AC implementation, transplant volume rose by 10%, with a 558% national allocation increase and a 34% local allocation decrease (p<0.001), with an increase in average distance from the donor hospital to the transplant center. Cold ischemic time increased from 5.74 to six hours (p<0.001), while the average waitlist time decreased from 232 to 182 days (p<0.001). High-severity patients received more transplants, with MELD scores rising from 23.1 to 24.7 (p<0.001).

Conclusions: The AC policy was associated with altered transplant allocation patterns, improved matching based on medical urgency, and reduced waitlist times. However, increased cold ischemic times and travel distances highlighted logistic challenges. While early trends show progress toward equity, further research is needed to address long-term effects and persistent geographic disparities.

## Introduction

Liver donation and transplantation in the United States are regulated by the United Network for Organ Sharing (UNOS), a private, non-profit organization that manages the Organ Procurement and Transplantation Network (OPTN), established by Congress in 1984 [1]. The primary goals of OPTN, ensured by the Final Rule in 1999, are to establish the framework for equitable donated organ matching, allocation, and distribution based on measurable medical criteria and geographic location [1].

Liver allocation has undergone several changes since its inception. Initially, it was based on hospitalization status and time on the waiting list. From 1998 to 2002, UNOS used the Child-Turcotte-Pugh (CTP) score to prioritize candidates, combining objective and measurable medical data with subjective assessments [1]. In 2002, the CTP score was replaced by the Model for End-Stage Liver Disease (MELD) score, which focused solely on measurable medical criteria: serum bilirubin, international normalized ratio (INR), and serum creatinine. In 2016, the MELD-Sodium score was introduced after research showed that hyponatremia (sodium (Na) <126 mEq/L) was a significant risk factor for the survival of liver cirrhosis patients [1]. In 2023, the MELD 3.0 score further refined the system by including factors such as patient sex at birth and serum albumin levels, addressing gender disparities in liver transplant access [2].

Prior to 2005, liver donations were offered first to patients with the highest MELD scores within the local donor-service area (DSA), then regionally, and finally nationally. However, this approach led to geographic disparities due to variations in population size, demographics, and available transplant facilities. UNOS implemented policy changes between 2005 and 2013 to address inequities, establishing priority levels based on MELD scores. Status 1 patients, who are critically ill with acute liver failure and have a life expectancy of seven days or less, were given the highest priority [3]. The next priority levels were (2) patients with MELD scores greater than 35, (3) patients within the DSA with MELD scores above 15, (4) regional patients with MELD scores above 15, (5) national patients with MELD scores above 15, and (6) regional patients with MELD scores below 15 [3].

Despite these efforts, geographic disparities persisted in MELD scores at transplant and waitlist mortality rates [3]. In 2020, the Acuity Circle (AC) model was implemented to further address these issues [1,4]. The policy established a zone-based distribution in which donor livers are offered to recipients based on distance in nautical miles (NM) from the donor hospital to the transplant hospital [5]. The donor liver is first offered to status 1 patients within 500 NM. The organ is then offered to the highest disease severity group based on MELD score thresholds in expanding geographic circles of 150 NM, then 250 NM, then 500 NM. If the organ is not accepted, then the next level of severity is considered in the same expanding distribution. This process is repeated for all severity thresholds greater than MELD 15 (MELD ≥ 37, MELD 33-36, MELD 29-32, MELD 15-28), after which the organ is offered nationally, then lastly offered to patients with MELD scores <15 within the geographic circles [3,6].

The AC model has shown early improvements in waitlist outcomes, particularly for high-MELD patients, but also raised concerns, such as increased travel costs and decreased transplantation for multivisceral candidates [7, 8]. Research is ongoing to evaluate the impact of this zone-based policy on overall transplant outcomes. The AC model has shown early improvements in waitlist outcomes, particularly for high-MELD patients, but also raised concerns, such as increased travel costs and decreased transplantation for multivisceral candidates [7,8]. Research is ongoing to evaluate the impact of this zone-based policy on overall transplant outcomes. This study aims to assess the early impact of the AC policy by comparing transplant metrics, donor characteristics, and recipient characteristics in the three years before and after implementation. Given the lack of national, retrospective studies spanning this full timeframe, we used a descriptive approach to explore changes in geographic distribution, disease severity, and allocation patterns.

## Materials and methods

We conducted a retrospective cohort study using data obtained from the National Standard Transplant Analysis and Research (STAR) file [9]. This database includes detailed information on all liver transplant waitlist registrations and transplant procedures in the United States since October 1, 1987, as reported to the OPTN. 

We included all liver transplant recipients who underwent transplantation in the three-year period before (February 4, 2017, to February 3, 2020) and after (February 4, 2020, to February 3, 2023) the implementation of the allocation policy.

We analyzed donor characteristics, including donation after brain death (DBD) versus donation after circulatory death (DCD), total cold ischemia time (hours), and use of expanded criteria grafts (ECG). Expanded criteria were defined according to institutional standards and typically included donors over the age of 60 or with infectious risk factors such as hepatitis B or C exposure.

We also analyzed recipient characteristics, including gender, ethnicity, BMI, primary diagnosis at listing, MELD and Pediatric End-Stage Liver Disease (PELD) scores, waitlist time (in days), and transplant location. Primary diagnoses included alcoholic cirrhosis, metabolic dysfunction-associated steatohepatitis (MASH) cirrhosis, hepatitis B and C-associated cirrhosis, acute alcohol-associated hepatitis, cryptogenic cirrhosis, and primary liver malignancy (hepatocellular carcinoma (HCC)). MELD and PELD scores were assessed at the time of transplant. Location was stratified by transplant region, state, and geographic category of liver allocation (local, regional, or national).

To assess allocation and travel impact, we calculated the average distance in NM between the donor hospital and transplant center pre- and post AC. We also evaluated changes in allocation type distribution (local, regional, and national) during the same periods.

Statistical analyses were performed using Jamovi software (v2.4.8; The jamovi project (2023)), which is built on the R statistical language (The R Core Team, R Foundation for Statistical Computing, Vienna, Austria). Categorical variables were compared using chi-squared tests. Continuous variables were analyzed using independent sample t-tests, and Welch’s t-tests were applied when Levene’s test indicated unequal variance. These univariate statistical methods are appropriate for descriptive comparisons but do not control for potential confounding variables. Significance was set at p < 0.05. The study adhered to Strengthening the Reporting of OBservational studies in Epidemiology (STROBE) guidelines for reporting observational studies [10].

## Results

Donor characteristics 

DBD or DCD

There was a statistically significant difference (p < .001) in the frequency of DBD compared to DCD. Pre-AC, there were 22,201 (92.6%) of DBD and 1,769 (7.4%) of DCD. Post AC, DCD usage increased to 2,799 (10.7%), while DBD accounted for 23,333 (89.3%) of accepted liver donations. 

Total Cold Ischemia Time

Average total cold ischemic time increased significantly from 5.74 hours (SD = 2.3) pre-AC to six hours (SD = 2.6) post AC (p < .001). 

ECG 

There was no statistically significant difference in the number of expanded criteria donor grafts pre- and post AC (p=0.184). Pre-AC, 5,230 (21.8%) of liver transplants used ECG, compared to 5,574 (21.3%) post AC.

Recipient characteristics

Demographics (Ethnicity, Gender, BMI)

Ethnicity: There was a statistically significant difference in the ethnicity of liver transplant recipients overall (p < .001). Table 1 displays demographics before and after AC implementation, with White recipients comprising the majority in both periods.

**Table 1:** Comparison of donor and recipient characteristics before and after Acuity Circle (AC) policy implementation Values are presented as n (%) or mean (SD), as appropriate. Only the primary diagnoses and states with the five greatest positive and five greatest negative changes in allocation percentage were included. P-values < 0.05 were considered statistically significant and are marked with an asterisk (*). The term “Non-Hispanic” is specified directly in the National Standard Transplant Analysis and Research (STAR) database and represents the underlying data structure. DBD: donation after brain death; DCD: donation after circulatory death; MASH: metabolic dysfunction-associated steatohepatitis; HCC: hepatocellular carcinoma; MELD: Model for End-Stage Liver Disease; PELD: Pediatric End-Stage Liver Disease; NM: nautical miles

Category	Variable	Pre-AC Policy	Post AC Policy	P-value
Donor characteristics	DBD	22201 (92.6%)	23333 (89.3%)	<0.001*
	DCD	1769 (7.4%)	2799 (10.7%)	<0.001*
	Total cold ischemia time (hours)	5.74 (2.28)	5.97 (2.55)	<0.001*
	Expanded criteria grafts (standard)	18740 (78.2%)	20558 (78.7%)	0.184
	Expanded criteria grafts (expanded)	5230 (21.8%)	5574 (21.3%)	0.184
Recipient characteristics	White, Non-Hispanic	17550 (69.4%)	19148 (68.9%)	<0.001*
	Black, Non-Hispanic	2148 (8.5%)	2047 (7.4%)	<0.001*
	Hispanic/Latino	4073 (16.1%)	4933 (17.7%)	<0.001*
	Asian, Non-Hispanic	1058 (4.2%)	1180 (4.2%)	<0.001*
	American Indian/Alaska Native, Non-Hispanic	225 (0.9%)	267 (1.0%)	<0.001*
	Native Hawaiian/other Pacific Islander, Non-Hispanic	56 (0.2%)	45 (0.2%)	<0.001*
	Multiracial, Non-Hispanic	168 (0.7%)	176 (0.6%)	<0.001*
	Male	16066 (63.6%)	17242 (62.0%)	<0.001*
	Female	9212 (36.4%)	10554 (38.0%)	<0.001*
	BMI (kg/m2)	28.1 (6.36)	28.3 (6.47)	<0.001*
Primary diagnosis	Alcoholic cirrhosis	5893 (30.5%)	7613 (40.3%)	<0.001*
	Alcohol-associated cirrhosis without acute alcohol-associated hepatitis	33 (0.2%)	1285 (6.8%)	<0.001*
	MASH cirrhosis	4277 (22.1%)	5211 (27.6%)	<0.001*
	Acute alcoholic hepatitis	285 (1.5%)	577 (3.1%)	<0.001*
	Acute alcohol-associated hepatitis with or without cirrhosis	7 (0.0%)	277 (1.5%)	<0.001*
	Cirrhosis: cryptogenic (idiopathic)	985 (5.1%)	922 (4.9%)	<0.001*
	Biliary atresia: extrahepatic	552 (2.9%)	496 (2.6%)	<0.001*
	Alcoholic cirrhosis with hepatitis C	643 (3.3%)	461 (2.4%)	<0.001*
	Primary liver malignancy (HCC + cirrhosis)	1671 (8.6%)	1427 (7.6%)	<0.001*
	Cirrhosis type C (hepatitis C-associated cirrhosis)	2818 (14.6%)	1431 (7.6%)	<0.001*
MELD/PELD scores	MELD	23.1 (10.8)	24.7 (10.6)	<0.001*
	PELD	10.7 (14.8)	11.0 (15.1)	0.581
Waitlist metrics	Time on waitlist (days)	232 (446)	182 (402)	<0.001*
Geographic distribution	Region 1	995 (3.9%)	1059 (3.8%)	<0.001*
	Region 2	2987 (11.8%)	2931 (10.5%)	<0.001*
	Region 3	4082 (16.1%)	4085 (14.7%)	<0.001*
	Region 4	2605 (10.3%)	2969 (10.7%	<0.001*
	Region 5	3940 (15.6%)	4608 (16.6%)	<0.001*
	Region 6	743 (2.9%)	789 (2.8%)	<0.001*
	Region 7	2018 (8.0%)	2325 (8.4%)	<0.001*
	Region 8	1610 (6.4%)	1694 (6.1%)	<0.001*
	Region 9	1375 (5.4%)	2011 (7.2%)	<0.001*
	Region 10	2459 (9.7%)	2721 (9.8%)	<0.001*
	Region 11	2464 (9.7%)	2604 (9.4%)	<0.001*
State (Top 10 only)	New York	1262 (5.0%)	1754 (6.3%)	<0.001*
	Florida	1654 (7.3%)	2027 (7.3%)	<0.001*
	Oklahoma	221 (0.9%)	407 (0.6%)	<0.001*
	Ohio	1155 (5.0%)	1389 (5.0%)	<0.001*
	California	2814 (11.2%)	3214 (11.6%)	<0.001*
	Georgia	867 (3.5%)	858 (3.1%)	<0.001*
	Alabama	383 (1.5%)	302 (1.1%)	<0.001*
	Louisiana	511 (2.0%)	432 (1.6%)	<0.001*
	Pennsylvania	1212 (4.8%)	1174 (4.2%)	<0.001*
	Arizona	729 (2.9%)	625 (2.3%)	<0.001*
Allocation type	Local	16451 (65.1%)	10827 (39.0%)	<0.001*
	Regional	7424 (29.4%)	7734 (27.8%)	<0.001*
	National	1403 (5.6%)	9234 (33.2%)	<0.001*
	Foreign donor	0 (0.0%)	1 (0.0%)	<0.001*
Distance	Donor to recipient (NM)	155 (234)	197 (229)	<0.001*

Gender: The gender of recipients saw statistically significant changes pre- and post AC (p<.001). Female transplant recipients increased while male recipients decreased in the three years post AC as demonstrated in Table 1. 

BMI: Recipient BMI was statistically increased with an average BMI of 28.1 kg/m^2^ (SD=6.4) pre-AC and a BMI of 28.3 kg/m^2 ^(SD=6.5) post AC (p<.001). 

Primary diagnosis at transplant listing

Alcoholic Cirrhosis 

Alcoholic cirrhosis as a primary diagnosis for liver transplantation occurred in a greater proportion of patients post AC. Pre-AC, 30.5% (n=5,893) of recipients had a primary diagnosis of alcoholic cirrhosis, while post AC, they made up 40.3% (n=7,613) of recipients transplanted (p<0.001).

MASH Cirrhosis 

There was a statistically significant increase in MASH cirrhosis as the primary diagnosis for transplant post AC. MASH cirrhosis made up 22.1% (n=4,277) of all liver transplant recipients pre-AC and increased to 27.6% (n=5,211) post AC (p<0.001). 

Cirrhosis Type C (Hepatitis C-associated Cirrhosis) 

There was a statistically significant decrease in liver transplant recipients with a primary diagnosis of hepatitis C-associated cirrhosis. Pre-AC, 14.6% (n=2,818) of recipients had this diagnosis, which decreased to 7.6% (n=1,431) of recipients post AC (p<.001). 

Primary Liver Malignancy (HCC + Cirrhosis) 

HCC in the setting of cirrhosis was listed as the primary diagnosis for transplantation in 8.6% (n=1,671) of liver transplant recipients pre-AC. Post AC, this diagnosis decreased to 7.6% (n=1,427) of recipients (p<0.001). 

Cirrhosis Type B (Hepatitis B-associated Cirrhosis)

Hepatitis B-associated cirrhosis as a primary diagnosis made up 1.5% (n=289) of recipients transplanted pre-AC and 1.2% (n=227) of recipients post AC, demonstrating a significant decrease (p<0.001). 

Acute Alcohol Associated Hepatitis With or Without Cirrhosis

Patients with a primary diagnosis of acute alcohol-associated hepatitis with or without cirrhosis made up 0.0% (n=7) of transplanted patients pre-AC. However, post policy change, this percentage increased to 1.5% (n=277) of transplanted patients (p<0.001). Similarly, patients with a primary diagnosis of acute alcoholic hepatitis increased from 1.5% (n=285) pre-AC to 3.1% (n=577) post policy change, an increase of 1.6% (p<0.001).

MELD/PELD score

MELD Score

The average MELD score in liver transplant recipients increased significantly from 23.1 (SD= 10.8) pre-AC to 24.7 (SD=10.6) post AC (p<0.001). Among the total recipients, 9,745 of 26,608 (36.6%) were in the high MELD category pre-AC, compared to 10,526 of 23,877 (44.1%) post AC, as shown in Table 2.

**Table 2:** Number of liver transplant recipients by MELD category before and after Acuity Circle (AC) policy implementation Values are presented as n (%). P-values < 0.05 were considered statistically significant and are marked with an asterisk (*). Table totals reflect only adult patients with MELD scores. Pediatric liver transplant recipients with PELD scores were analyzed separately and are excluded from this table. MELD: Model for End-Stage Liver Disease; PELD: Pediatric End-Stage Liver Disease

MELD Categories	Score Range	Number of Transplant Recipients Pre-AC	Number of Transplant Recipients Post AC	P-value
High	>31	9745 (36.6%)	10526 (44.1%)	<0.001*
Intermediate	21-30	8631 (32.4%)	7029 (29.4%)	<0.001*
Low	<20	8232 (30.9%)	6322 (28.5%)	<0.001*
Total	-	26608	23877	-

PELD Score

The average PELD score of pediatric recipients transplanted pre-AC was 10.7 (SD=14.8), increased to 11 (SD=15.1) post AC (P=0.581).

Time on waitlist

The length of time recipients spent on the transplant list significantly decreased post AC (p<0.001). Pre-AC recipients overall spent an average of 232 days (SD=446) on the waitlist compared to an average of 182 days (SD = 402) post AC. 

**Figure 1:**
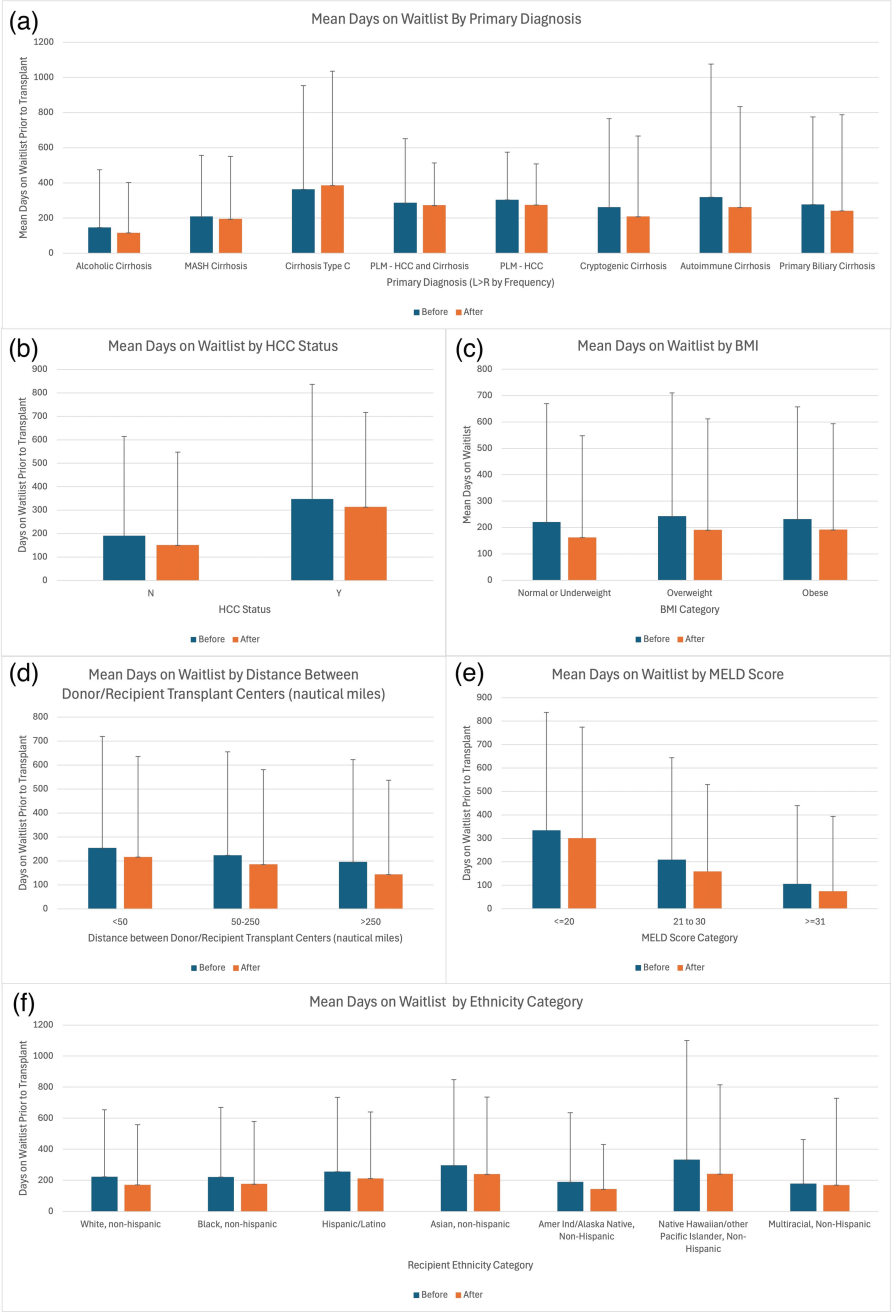
Mean days on the liver transplant waitlist before and after the allocation policy change, stratified by key clinical and demographic variables. Subfigures show (a) primary diagnosis, (b) HCC status, (c) donor–recipient center distance, (d) BMI category, (e) MELD score category, and (f) ethnicity category. “Before” and “After” refer to time periods prior to and following policy implementation. Error bars represent one standard deviation. The term “Non-Hispanic” is specified directly in the National Standard Transplant Analysis and Research (STAR) database and represents the underlying data structure. MASH:  metabolic dysfunction-associated steatohepatitis; HCC: hepatocellular carcinoma; PLM: primary liver malignancy; MELD: Model for End-Stage Liver Disease

We further characterized the time that transplant recipients spent on the liver waitlist based on characteristics of the recipient, as illustrated in Figure 1. 

All liver transplant recipients had shorter wait times on the transplant list after the liver allocation policy change. (p<0.001). However, when we stratified waitlist times by MELD score, HCC status, recipient BMI, distance, and primary diagnosis, no individual subgroup showed a statistically significant change. This may reflect modest decreases across all subgroups that, though individually non-significant, cumulatively contributed to the overall significant reduction in waitlist time, as shown in Table 3.

**Table 3:** Sub-group analysis of waitlist times and distance between donor/recipient Values are formatted as n (%) or mean (SD), as appropriate. P-values < 0.05 were considered statistically significant and are marked with an asterisk (*). P-values represent differences in outcomes between pre- and post-AC policy periods within each subgroup. The term “Non-Hispanic” is specified directly in the National Standard Transplant Analysis and Research (STAR) database and represents the underlying data structure. AC: Acuity Circle; MASH:  metabolic dysfunction-associated steatohepatitis; HCC: hepatocellular carcinoma; PLM: primary liver malignancy; MELD: Model for End-Stage Liver Disease

Category	Subgroup	Pre-AC	Post AC	P-value
Waitlist: MELD	≤ 20	334 (503)	301 (470)	0.092
-	20-30	209 (453)	159 (370)	0.092
-	≥ 30	106 (334)	74.4 (319)	0.092
Waitlist: HCC status	Yes	191 (423)	151 (396)	0.519
-	No	348 (488)	314 (403)	0.519
Waitlist: BMI	Normal or underweight	221 (448)	163 (385)	0.096
-	Overweight	243 (467)	191 (421)	0.096
-	Obese	232 (425)	192 (401)	0.096
Waitlist: Distance	<50	254 (465)	217 (419)	0.303
-	50-250	224 (431)	186 (394)	0.303
-	>250	196 (426)	144 (393)	0.303
Waitlist: Diagnosis	Alcoholic cirrhosis	147 (327)	117 (285)	0.221
-	MASH cirrhosis	209 (348)	195 (356)	0.221
-	Cirrhosis type C	364 (589)	386 (649)	0.221
-	PLM-HCC and cirrhosis	287 (364)	273 (240)	0.221
-	PLM-HCC	304 (271)	275 (233)	0.221
-	Cryptogenic cirrhosis	262 (504)	209 (457)	0.221
-	Autoimmune cirrhosis	320 (756)	262 (572)	0.221
-	Primary biliary cirrhosis	278 (497)	242 (545)	0.221
Waitlist: Ethnicity	White, Non-Hispanic	224 (431)	172 (387)	0.426
-	Black, Non-Hispanic	222 (448)	177 (402)	0.426
-	Hispanic/Latino	257 (477)	213 (427)	0.426
-	Asian, Non-Hispanic	297 (551)	240 (497)	0.426
-	American Indian/Alaska Native, Non-Hispanic	190 (446)	144 (287)	0.426
-	Native Hawaiian/other Pacific Islander, Non-Hispanic	333 (767)	242 (573)	0.426
-	Multiracial, Non-Hispanic	179 (284)	170 (558)	0.426
Distance: MELD	≤ 20	139 (240)	168 (259)	<0.001*
-	20-30	127 (206)	176 (203)	<0.001*
-	≥ 30	183 (209)	232 (180)	<0.001*

Location

Overall, there was an increase in total transplants, with 25,278 transplants performed in three years pre-AC and 27,796 transplants performed in three years post AC. These totals include both adult and pediatric liver transplant recipients. Some subgroup analyses (e.g., Table 2) only include adult recipients with MELD scores, excluding pediatric patients with PELD scores due to the use of a different scoring system. There is a statistically significant difference between local, regional, and national allocation of liver transplantation (p<.001). Local allocation decreased from 16,451 (65.1%) pre-AC to 10,827 (39.0%) post AC, while national allocation decreased from 1,403 (5.6%) pre-AC to 9,234 (33.2%) post AC, reflecting the intended redistribution of donor livers under the AC policy.

**Figure 2:**
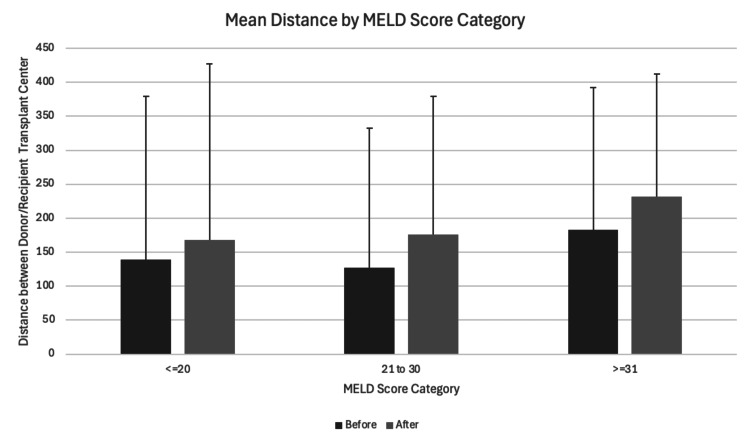
Mean distance between donor and recipient transplant centers by MELD score category Error bars depict one standard deviation. MELD: Model for End-Stage Liver Disease

The recorded average distance from donor hospitals to transplant centers saw a statistically significant increase from a mean distance of 155 NM (SD=234) to 197 NM (SD=229) pre- and post AC period (p<0.001). Among low (n=8,232 pre-AC; n=6,322 post-AC), intermediate (n=8,631; n=7,029), and high MELD score recipients (n=9,745; n=10,526), the average distance also rose significantly across all MELD scores, as shown in Figure 2 and Table 3. 

Furthermore, following the shift away from regional and DSA-based allocation, the number of liver transplants across UNOS regions demonstrated statistically significant variation (p < .001). Region 2 was the only region to experience a decline, with a 2% reduction in transplant volume. In contrast, Regions 5 and 9 experienced the greatest increases, with transplant volumes rising by 17% and 46%, respectively, suggesting substantial regional redistribution of transplant activity post policy implementation.

On the regional level, the median MELD score at the time of transplant increased across all UNOS regions following the implementation of the AC policy. As shown in Table 4, Regions 1, 2, 3, 4, 5, 6, 7, and 11 experienced the largest increases in median MELD scores, with Region 1 demonstrating the greatest increase from 21 to 27 (+6 points). This was followed by Regions 2, 9, and 11, which all showed increases of +4 points. Regions 3, 4, and 6 had more modest increases ranging from +2 to +3. Region 10 remained stable with no change in median MELD score. While these trends suggest a shift toward transplanting sicker patients in several regions, the substantial overlap in standard deviations (approximately 13.6 to 16.6 across regions) indicates that these differences were not statistically significant. These findings suggest that while regional variation exists, it is likely due to natural variability rather than a uniform effect of the AC policy.

**Table 4:** Median MELD scores by UNOS Region pre- and post AC policy implementation MELD: Model for End-Stage Liver Disease; UNOS: United Network for Organ Sharing; AC: Acuity Circle

Region	Pre-AC Median	Post AC Median	Change in Median
1	21	27	6
2	22	26	4
3	22	24	2
4	23	26	3
5	24	25	1
6	23	25	2
7	23	25	2
8	22	23	1
9	22	26	4
10	21	21	0
11	22	26	4

**Figure 3:**
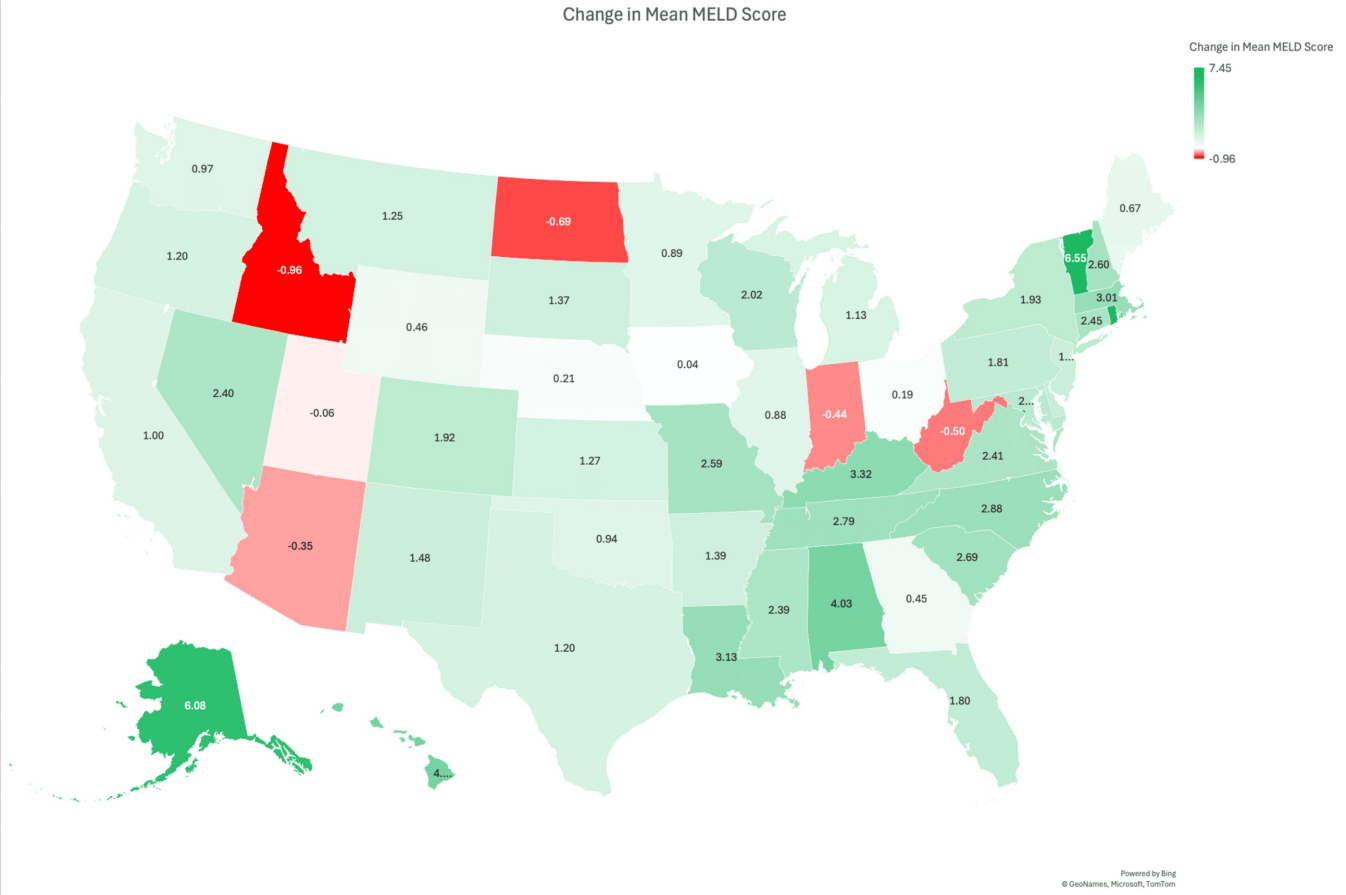
Change in median MELD score at the time of liver transplant by U.S. state before and after Acuity Circle policy implementation. Green shades represent increases; red shades represent decreases. Data reflect the difference in median MELD scores by state. MELD: Model for End-Stage Liver Disease Image credit: Created by the authors (Daniel Sculley). Data source: STAR file, Scientific Registry of Transplant Recipients (SRTR).

On the state level, the severity of illness at the time of transplant, as measured by the median MELD score, showed substantial variation between states following the implementation of the AC policy. As shown in Figure 3, the largest increases in median MELD scores occurred in Alaska (+13.0), Rhode Island (+11.0), and the District of Columbia (+11.0), suggesting that sicker patients were more likely to receive transplants in these states post policy change. Conversely, North Dakota (-3.0), Idaho (-2.0), and Montana (-1.0) experienced declines in median MELD scores. However, due to considerable overlap in standard deviations (ranging from approximately 14 to 16 across states), these differences were not statistically significant. This suggests that the observed changes are more likely due to random variation rather than a uniform shift driven by the AC policy. Supporting this, the standard deviation of median MELD scores between states was 2.7 pre-AC and 2.2 post AC, and between regions was 0.9 pre-AC and 1.7 post AC. This range of variability suggests that while some geographic differences exist, they are likely not meaningful enough to indicate consistent, policy-driven change.

## Discussion

The AC policy was implemented to address geographic disparities in liver transplant access and improve waitlist outcomes by prioritizing disease severity over geographic boundaries [9]. In removing regional and DSA constraints, this zone-based allocation model expanded national organ sharing and emphasized medical urgency [11]. Our findings demonstrate that the AC model has achieved some of its intended goals, particularly in improving access for high-acuity patients and reducing overall waitlist times, though it introduced logistical challenges that warrant continued evaluation.

This study showed that post AC, there was an overall increase in the total volume of liver transplants and a tremendous increase in national allocation by 558% from pre-AC. With more national allocation and only a 4% regional increase, the data underline the significant increase in distance traveled from the donor hospital to the transplant center. Under this policy, patients are gaining greater access to donors' livers that they may not have had previously; however, there is still a varied impact across the nation [11]. This could be attributed to the number of transplant centers accessible in rural vs. urban communities and across state lines. Patients with higher MELD scores mainly saw an increase in transplantation post AC despite distance from the transplant center. On average, the distance from the donor hospital to the transplant center increased by 49 NM for patients with MELD scores≥30. Furthermore, there was a significant decrease in patients' time on the liver waitlist post AC, decreasing by 50 days from pre-AC to post AC. In addition, patients with MELD across all levels of severity experienced decreased waitlist time. Longer wait times have been associated with greater adverse outcomes post‑transplant (as shown by evidence of significant mortality reductions when waitlists were shortened and acuity-based prioritization was implemented) [12].

Despite the observed trends of increased median MELD scores across states and regions following the implementation of the AC policy, our analysis suggests that these changes were not statistically significant. While mean MELD scores rose modestly in most UNOS regions and certain states demonstrated more pronounced changes, the standard deviation of median MELD scores between states was 2.7 pre-AC and 2.2 post AC, and between regions was 0.9 pre-AC and 1.7 post AC. These overlapping ranges suggest that the observed variation is likely due to natural variability rather than a uniform or significant shift driven by policy change alone. This finding highlights that although AC implementation aimed to reduce geographic disparities in transplant access, measurable differences in disease severity at transplant across states and regions appear relatively unchanged within the first three years following AC implementation. Longer follow-up and additional analyses may be necessary to detect meaningful geographic trends.

An analysis of primary diagnoses for a liver transplant at listing revealed a significant shift in pre- and post AC policy implementation. The proportion of patients listed with HCC as their primary diagnosis decreased by 1.5% (from 8.6%, n=1,671 to 7.6%, n=1,427) following the policy change. Concurrently, alcoholic cirrhosis increased from 30.5% (n=5,893) to 40.3% (n=7,613), and MASH cirrhosis rose from 22.1% (n=4,277) to 27.6% (n=5,211). Similarly, acute alcohol-associated hepatitis increased from 1.5% (n=285) to 3.1% (n=577), and acute alcoholic hepatitis with or without cirrhosis increased from 0.0% (n=7) to 1.5% (n=277). These findings reflect changing trends in disease prevalence and the prioritization of specific diagnoses within the transplant population. Of note, these changes align with current disease trends, which have shown the incidence of MASH to have increased from 1.51% in 2010 to 2.79% in 2020, with an expected continued upward trend of MASH over the current decade [13]. 

Various donor characteristics were also compared, including the type of donor, total cold ischemic time, and expanded criteria donors. Historically, the majority of liver donations have been from DBD, given that DCD has been associated with worse outcomes, partly attributable to the ischemic injury that solid organs suffer during the transplantation process [14]. Though DBD is still the dominant type of donor, the AC model has increased the amount of DCD organs utilized for liver transplantation. Although this trend may result from multiple factors, it can be partially attributed to the advancements made with normothermic machine perfusion and normothermic regional perfusion. Other studies have shown that centers in non-high-population states under the AC model significantly increased DCD utilization by 155%, suggesting the greater need to evaluate the outcomes of DCD and how center-based usage has changed since 2020 [15]. Total cold ischemic time increased after the liver allocation policy change, which can be explained by the increased distance that a donor's liver travels. These results highlight the complex relationship between donor characteristics, ischemic injury, and the ongoing challenges in improving transplant outcomes. 

While this study aims to explore the changes in liver allocation and transplantation post AC policy, it is essential to acknowledge the limitations that may impact findings and interpretations. The post AC policy patient population received liver transplants between February 4, 2020, and February 3, 2023. This period was significantly influenced by the COVID-19 pandemic, which profoundly affected various aspects of healthcare, including organ transplantation. While we lack comprehensive data to quantify the specific impact of COVID-19 during this time, it undeniably played a crucial role in shaping healthcare dynamics. Several mechanisms may have contributed. Flight restrictions and staffing shortages likely increased transport times. Operating room closures and limited ICU beds may have changed center acceptance practices, favoring sicker patients. Delayed outpatient care may have reduced referrals for HCC while increasing listings for alcohol-related disease. Shifts in donor availability and the use of machine perfusion may also have influenced donor use. Because our study was descriptive, we cannot separate these effects from the impact of the AC policy.

Additionally, we did not control for underlying temporal trends that may have been occurring independently of the policy change. Ongoing shifts in disease prevalence, referral patterns, transplant center behavior, or other secular trends may have contributed to some of the observed differences. This limitation makes it difficult to attribute changes solely to the AC policy. Moreover, although we observed a statistically significant reduction in overall waitlist time, no individual subgroup reached significance. This discrepancy may reflect modestly distributed changes across categories or insufficient power in subgroup analyses. Our analyses used univariate methods that do not account for confounding. A multivariable regression model would be a more suitable approach to adjust for overlapping factors such as demographics, donor type, and regional variation. Future studies should use this approach to better isolate the independent effect of the AC policy.

Furthermore, we were unable to evaluate the effect of the AC policy change on individual transplant centers due to the limitations of the National STAR database. This would provide valuable insights into how the policy change has impacted large vs. small healthcare institutions. In addition, the recipient's diagnosis reflects the patient’s diagnosis at the time of the listing rather than at the time of transplantation. Patients can often develop compounding liver pathologies, which can affect the patient’s MELD score and, consequently, the urgency with which they receive a liver. Without clarity on the diagnosis that necessitated liver transplantation at the time of the procedure, our interpretations may be flawed.

## Conclusions

As the liver allocation policy and the criteria for liver transplantation continue to evolve, ongoing research is needed. This research study examines the three years before and after the allocation policy change, but having a longer-term view of five or even 10 years after the implementation of this policy could provide more information about its impacts. With the increased distance that livers are being transported comes increased costs. Further analysis can be done to explore how the increasing demand for transport has transformed organ procurement organization practices. The financial burden this places on the patients and the healthcare system could be investigated in more depth. It is essential to consider how these changes may have impacted the equity of the transplants in the setting of geographic and socioeconomic disparities, which is crucial to consider when thinking about future policy improvements. Importantly, this study is exploratory and does not aim to prove causation. Future studies using multivariable regression and longer follow-up are necessary to confirm whether observed trends can be attributed directly to the AC policy.

## References

[1] Latt NL, Niazi M, Pyrsopoulos NT (2022). Liver transplant allocation policies and outcomes in United States: a comprehensive review. World J Methodol.

[2] Kim WR, Mannalithara A, Heimbach JK (2021). Meld 3.0: the Model for End-Stage Liver disease updated for the modern era. Gastroenterology.

[3] Polyak A, Kuo A, Sundaram V (2021). Evolution of liver transplant organ allocation policy: current limitations and future directions. World J Hepatol.

[4] Chyou D, Karp S, Shah MB, Lynch R, Goldberg DS (2021). A 6-month report on the impact of the Organ Procurement and Transplantation Network/United Network for Organ Sharing Acuity Circles policy change. Liver Transpl.

[5] (2025). System notice: Liver and intestinal organ distribution based on acuity circles implemented Feb. 4. https://unos.org/news/system-implementation-notice-liver-and-intestinal-organ-distribution-based-on-acuity-circles-implemented-feb-4/.

[6] Pawlak N, Song C, Alvi S (2023). Perceptions and early outcomes of the Acuity Circles allocation policy among liver transplant centers in the United States. Transplant Direct.

[7] Shimada S, Yoshida A, Abouljoud M (2025). Post-transplant outcomes and financial burden of donation after circulatory death donor liver transplant after the implementation of acuity circle policy. Clin Transplant.

[8] Ivanics T, Vianna R, Kubal CA (2022). Impact of the acuity circle model for liver allocation on multivisceral transplant candidates. Am J Transplant.

[9] (2025). OPTN/SRTR annual data reports. https://srtr.transplant.hrsa.gov.

[10] von Elm E, Altman DG, Egger M, Pocock SJ, Gøtzsche PC, Vandenbroucke JP (2008). The Strengthening the Reporting of Observational Studies in Epidemiology (STROBE) statement: guidelines for reporting observational studies. J Clin Epidemiol.

[11] Yilma M, Dalal N, Wadhwani SI, Hirose R, Mehta N (2023). Geographic disparities in access to liver transplantation. Liver Transpl.

[12] Giorgakis E, Ivanics T, Wallace D (2023). Acuity circles allocation policy impact on waitlist mortality and donation after circulatory death liver transplantation: a nationwide retrospective analysis. Health Sci Rep.

[13] Pierotti J (2025). The economic benefits of detecting liver disease early. https://lucemhealth.com/blog/economic-benefits-detecting-liver-disease-early/.

[14] Callaghan CJ, Charman SC, Muiesan P, Powell JJ, Gimson AE, van der Meulen JH (2013). Outcomes of transplantation of livers from donation after circulatory death donors in the UK: a cohort study. BMJ Open.

[15] Bekki Y, Myers B, Tomiyama K, Melcher ML, Sasaki K (2023). The impact of geographic location versus center practice on center volume in liver transplantation after the acuity circle policy. Clin Transplant.

